# Author Correction: Bridging of host-microbiota tryptophan partitioning by the serotonin pathway in fungal pneumonia

**DOI:** 10.1038/s41467-024-48040-7

**Published:** 2024-04-26

**Authors:** Giorgia Renga, Fiorella D’Onofrio, Marilena Pariano, Roberta Galarini, Carolina Barola, Claudia Stincardini, Marina M. Bellet, Helmut Ellemunter, Cornelia Lass-Flörl, Claudio Costantini, Valerio Napolioni, Allison K. Ehrlich, Cinzia Antognelli, Massimo Fini, Enrico Garaci, Emilia Nunzi, Luigina Romani

**Affiliations:** 1 Department of Medicine and Surgery, University of Perugia, Perugia, Italy; 2 Istituto Zooprofilattico Sperimentale dell’Umbria e delle Marche “Togo Rosati,”, Perugia, Italy; 3 CF Centre, Medical University Innsbruck, Innsbruck, Austria; 4 Division of Hygiene and Medical Microbiology, Innsbruck Medical University, Innsbruck, Austria; 5 School of Biosciences and Veterinary Medicine, University of Camerino, Camerino, Italy; 6 Department of Environmental Toxicology, University of California, Davis, CA USA; 7 University San Raffaele and Istituto di Ricovero e Cura a Carattere Scientifico (IRCCS) San Raffaele, Rome, Italy

**Keywords:** Fungal infection, Cystic fibrosis, Microbiome

Correction to: *Nature Communications* 10.1038/s41467-023-41536-8, published online 16 September 2023

The original version of this Article contained an error in Fig. 9, in which a duplicated microscopy image was present in panel f. This has been corrected in both the PDF and HTML versions of the Article.

