# Supplementary material for: Bridging of host-microbiota tryptophan partitioning by the serotonin pathway in fungal pneumonia
Source: Nat Commun. 2023 Sep 16;14:5753. doi: 10.1038/s41467-023-41536-8 (PMC10505232; doi:10.1038/s41467-023-41536-8)
Supplement: Supplementary file 1 — Supplementary Information [file 41467_2023_41536_MOESM1_ESM.pdf]

Supplementary Figures and Tables

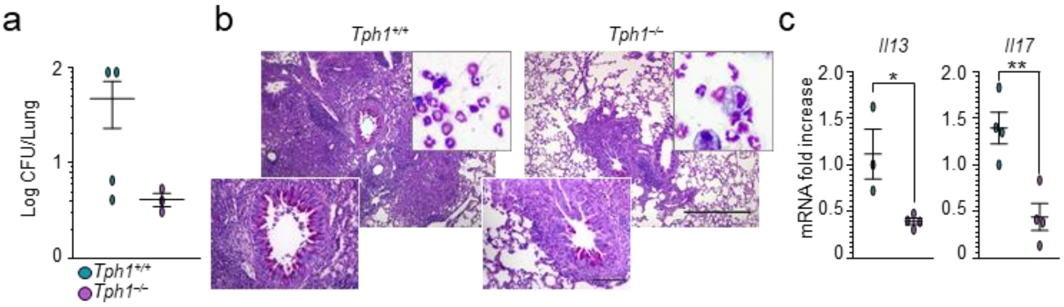

Figure S1. ***5-HT contributes to the development of fungal allergy*.** *Tph1*$^{+/+}$ and *Tph1*$^{-/-}$ mice, sensitized with crude culture filtrate fungal antigens, were challenged with *A. fumigatus* conidia and assessed at 7 dpi for (**a**) fungal growth, (**b**) lung histology (periodic acid-Schiff staining and, in the insets, cellular morphology in the bronco-alveolar lavage) and (**c**) *Il13* and *Il17* gene expression in lung (RT-PCR). Photographs were taken with a high-resolution microscope (Olympus BX51), 10X and 40X magnification (scale bars, 500 µm and 100 µm, respectively). Data are presented as mean ± SEM and representative of two experiments. The in vivo groups consisted of 4 mice/group. *p < 0.05 and **p < 0.01, *Tph1*$^{-/-}$ *vs* C57BL/6 mice. Unpaired t-test. Source Data are provided as a Source Data file.

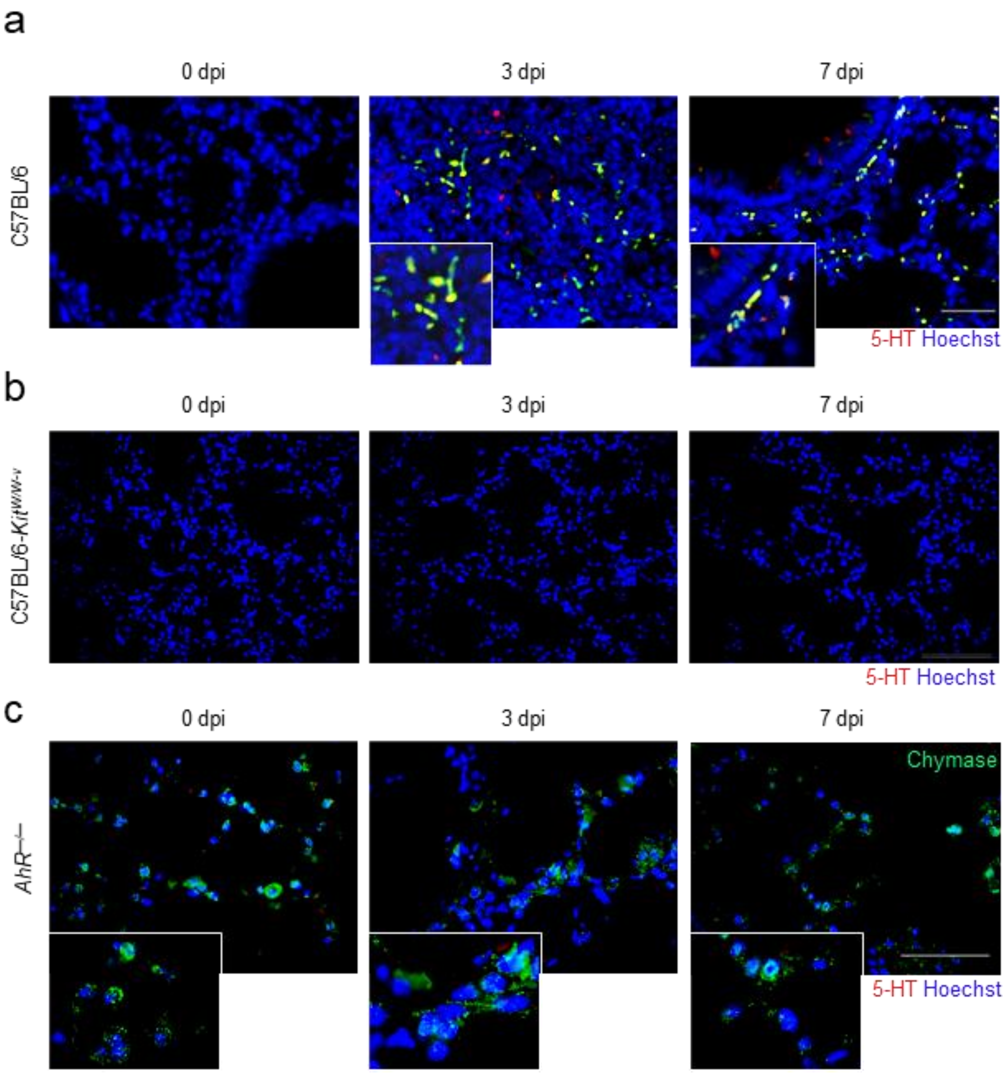

Figure S2. ***Mast cells produce 5-HT in the lung via AhR*****.** C57BL/6, MC-deficient C57BL6-*Kit*$^{W/W\text{-}v}$ and *AhR*$^{-/-}$ mice were infected intranasally with *A. fumigatus* and assessed at 3 or 7 days post-infection (dpi) for lung immunofluorescence of (**a**) CD41 and 5-HT, (**b**) 5-HT and (**c**) 5-HT and chymase (magnified in the insets) in perfused lungs. Hoechst was used for nuclear counterstain in blue. Photographs were taken with a high-resolution microscope (Olympus BX51), 40X and 100X magnification (scale bars 100 µm and 20 µm, respectively).

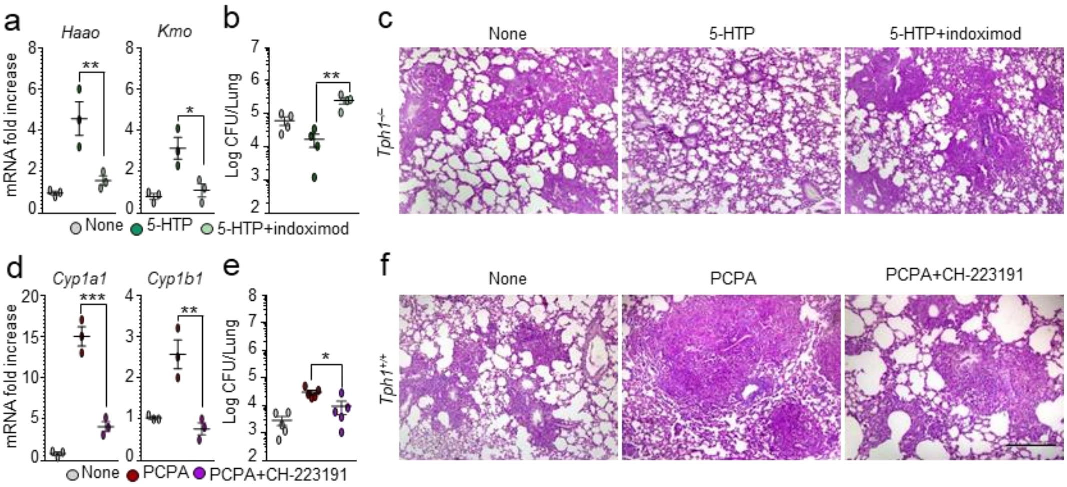

Figure S3. ***Effects of PCPA and 5-HTP administration in infection upon IDO1 or AhR inhibition, respectively*****.** (**a**-**c**) *Tph1*$^{-/-}$ mice were infected intranasally with *A. fumigatus* and treated intraperitoneally with 5-HTP (5.5 mg/mouse) in the presence or absence of indoximod (2 mg/mouse), one day before the infection and every other day post infection (dpi). (**d**-**f**) *Tph1*$^{+/+}$ mice were infected intranasally and treated intraperitoneally with PCPA (5.5 mg/mouse) in the presence or absence of CH-223191 (0.2 mg/mouse), as above. Mice were assessed at 7 dpi for the expression of Ido1- (**a**) and AhR- (**d**) related genes, fungal burden (**b** and **e**) and lung histology (periodic acid-Schiff staining) (**c** and **f**). Photographs were taken with a high-resolution microscope (Olympus BX51), 20X magnification (scale bar 200 µm). Data are expressed as mean ± SEM and representative from two experiments. *p < 0.05, **p < 0.01 and ***p < 0.001. 5-HTP+indoximod *vs* 5-HTP, PCPA+CH-223191 *vs* PCPA. The in vivo groups consisted of 5 mice/group. One-way ANOVA, Bonferroni post-hoc test. Source Data are provided as a Source Data file.

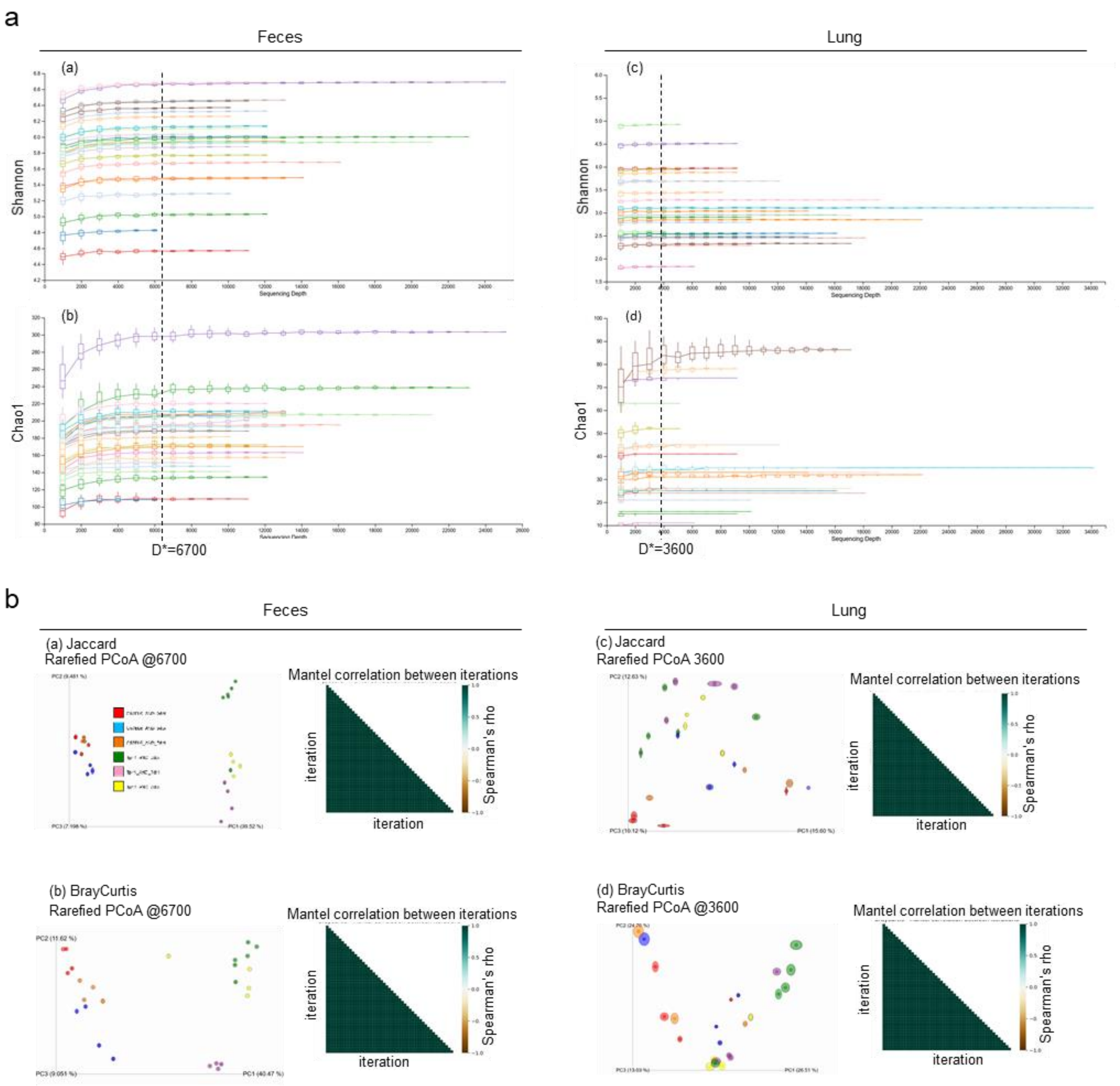

Figure S4. ***The rarefaction bands of diversities values at the chosen sequencing depth remain separated for each sample at sequence variant level for both feces and lungs*.** (**a**) Alpha rarefaction curves reach the plateau in both feces [panels (a) and (b)] and lungs [panels (c) and (d)]. Indexes have been evaluated from 50 feature tables rarefied at multiple sampling depths and boxplot of each sequenced sample vs the corresponding sequencing depth is shown. Median values of consecutive boxplots of each sample are connected with a line. Dashed-black line indicate the lowest number of reads taken in a sample. Shannon index [panels (a) and (c)] provides indications on richness and evenness of each sample. Chao1 [panels (b) and (d)] considers the contribution of rares to richness per sample. (**b**) PCoA plot of beta diversities evaluated on 50 rarefied feature tables in both feces [panels (a) and (b)] and lungs [panels (c) and (d)]. Jackknified PCoA plots of the first three components of diversity metrics are shown. Diversity indexes have been evaluated from 50 feature tables rarefied at 6700 for feces and at 3600 for lungs. Jaccard index [panels (a) and (c)] provides qualitative indications on the type of feature in each sample. Bray-Curtis index [panels (b) and (d)] considers the quantitative contribution of features per sample. Also, Mantel correlation (Spearman) test between rarefaction trials of each diversity matrix is shown for comparing the 50 rarefied diversities outcomes. Correlation values for all trials is close to 1 for all cases.

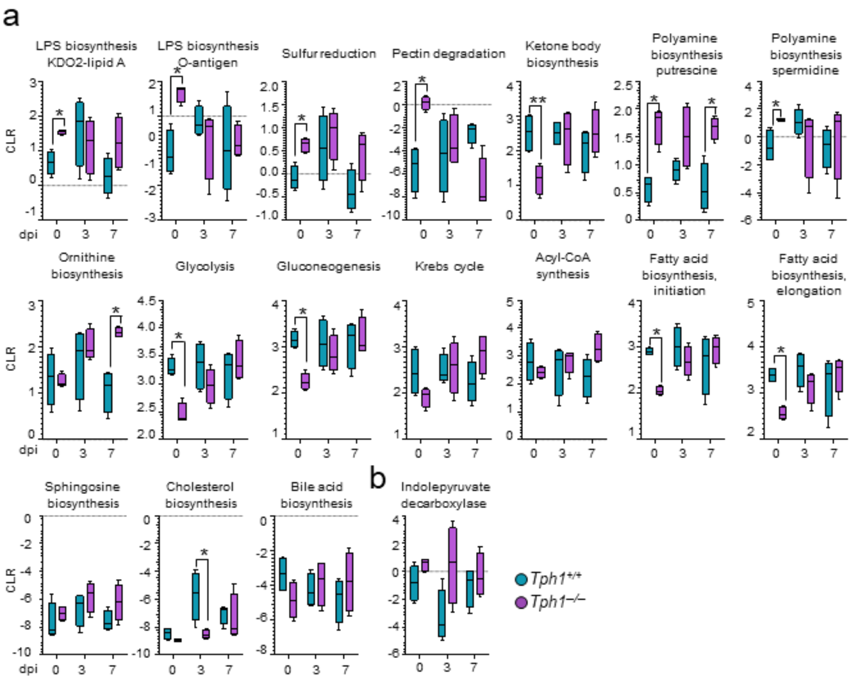

Figure S5. ***The microbiota functional activity is altered in the lung of Tph1$^{-/-}$ mice.*** Boxplots of (**a**) metabolic functions and (**b**) enzymatic activity (inferred by PICRUSt2 analysis and KEGG function and enzyme) in *A. fumigatus*-infected *Tph1*$^{+/+}$ or *Tph1*$^{-/-}$ mice at 3 and 7 days post-infection (dpi). Data are expressed as mean ± SEM and are representative from two experiments. *p < 0.05, *Tph1*$^{-/-}$ *vs Tph1*$^{+/+}$ mice. The in vivo groups consisted of 4 mice/group. Multiple t-tests. CLR, centered log.ratio. Source Data are provided as a Source Data file.

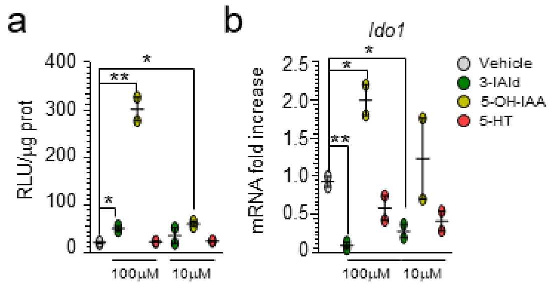

Figure S6. ***Activation of Ido1 by 5-OH-IAA.*** (**a**) AhR activity by luciferase assay and (**b**) Ido1 expression by RT-PCR in mouse hepatoma H1L6.1c3 cells treated with different doses (100/10 µM) of 3-IAld, 5-OH-IAA and 5-HT for 24h. Data are expressed as mean ± SEM and representative from two experiments. $*p < 0.05$ and $**p < 0.01$, treated *vs* untreated. Unpaired t-test. Vehicle, PBS alone. Source Data are provided as a Source Data file.

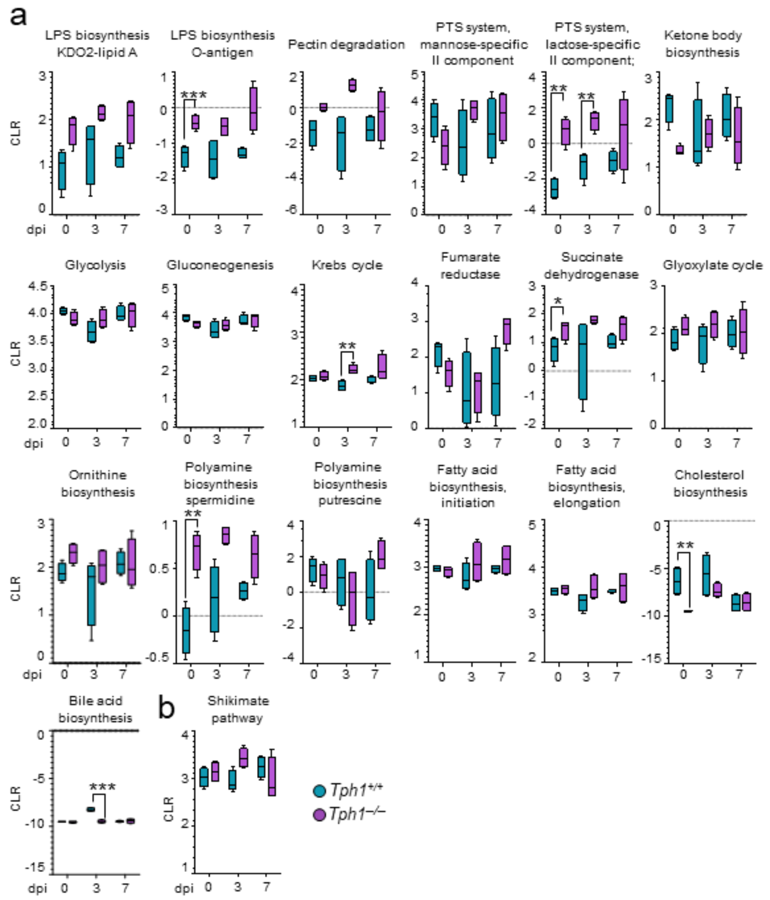

Figure S7. ***The microbiota functional activity is altered in the feces of Tph1$^{-/-}$ mice.*** Boxplots of (**a**) metabolic functions and (**b**) enzymatic activity (inferred by PICRUSt2 analysis and KEGG function and enzyme) in *A. fumigatus*-infected *Tph1*$^{+/+}$ or *Tph1*$^{-/-}$ mice at 3 and 7 days post-infection (dpi). Data are expressed as mean ± SEM and are representative from two experiments. *p < 0.05, **p < 0,01 and ***p < 0.001, *Tph1*$^{-/-}$ *vs Tph1*$^{+/+}$ mice. The in vivo groups consisted of 4 mice/group. Multiple t-tests. CLR, centered log.ratio. Source Data are provided as a Source Data file.

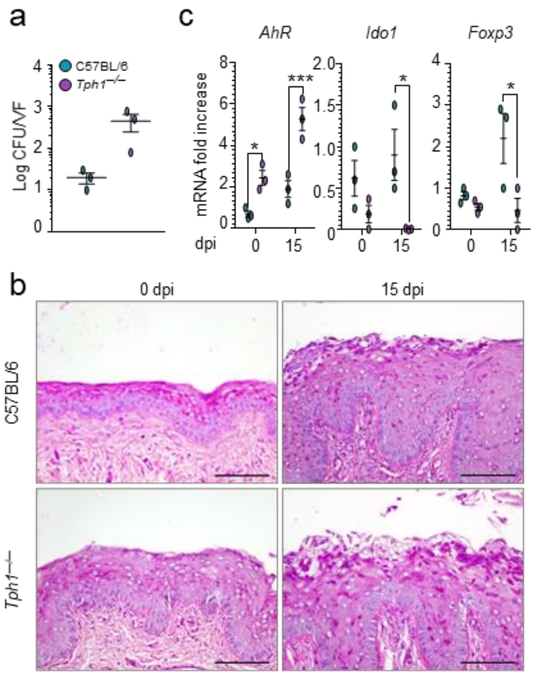

Figure S8. ***$Tph1^{-/-}$* mice are susceptible to vulvovaginal candidiasis.** C57BL/6 and *$Tph1^{-/-}$* mice were inoculated intravaginally with *Candida albicans* and assessed at 15 days post-infection (dpi) for (**a**) local fungal growth, (**b**) vaginal pathology (periodic acid-Shiff-staining) and (**c**) *AhR*, *Ido1* and *Foxp3* expression in vaginal tissues. Data are presented as mean ± SEM and representative of two experiments. The in vivo groups consisted of 3 mice/group. *$p < 0.05$ and ***$p < 0.001$, *$Tph1^{-/-}$ vs* C57BL/6 mice. Two-way ANOVA, Sidak post test. Source Data are provided as a Source Data file.

	0 dpi	3 dpi	Adjusted p value
IL-1β	ns	*	0.0146
TNF-α	ns	***	0.0006
GM-CSF	ns	ns	ns
CXCL1	ns	**	0.0059
CCL2	ns	*	0.0270
MIP-2	ns	**	0.0098
CCL7	ns	**	0.0100
CCL3	ns	*	0.0204
CCL4	ns	**	0.0010
CCL5	ns	*	0.0360
CCL11	ns	ns	ns
CXCL10	ns	*	0.0123

Table S1. Two-way ANOVA, Sidak post-hoc test. *p < 0.05, **p < 0.01, and ***p < 0.001, *Tph1*$^{-/-}$ *vs Tph1*$^{+/+}$ mice. ns, not statistically significant. Source Data are provided as a Source Data file.

IDO1	*Tph1*$^{-/-}$ 0 dpi *vs* *Tph1*$^{+/+}$ 0 dpi	ns	ns
	Tph1$^{-/-}$ 7 dpi *vs* *Tph1*$^{+/+}$ 7 dpi	**	0.0079
AhR	*Tph1*$^{-/-}$ 0 dpi *vs* *Tph1*$^{+/+}$ 0dpi	ns	ns
	Tph1$^{-/-}$ 7 dpi *vs* *Tph1*$^{+/+}$ 7 dpi	***	0.0004
5-HT	*Tph1*$^{-/-}$ 0 dpi *vs* *Tph1*$^{+/+}$ 0 dpi	****	< 0.0001
	Tph1$^{-/-}$ 3 dpi *vs* *Tph1*$^{+/+}$ 3 dpi	****	< 0.0001
	Tph1$^{-/-}$ 7 dpi *vs* *Tph1*$^{+/+}$ 7 dpi	*	0.0230

Table S2. Quantification of the corresponding average immunofluorescence intensities of IDO1, AhR and 5-HT. Two-way ANOVA, Bonferroni post-hoc test. **p < 0.01, ***p < 0.001 and **** p < 0.0001 *Tph1*$^{-/-}$ vs *Tph1*$^{+/+}$ mice. ns, not statistically significant. Adjusted P values: 0.0079 (IDO1, 7 dpi, *Tph1*$^{+/+}$ vs *Tph1*$^{-/-}$), 0.0004 (AhR, 7 dpi, *Tph1*$^{+/+}$ vs *Tph1*$^{-/-}$), < 0.0001 (5-HT, 0 dpi, *Tph1*$^{+/+}$ vs *Tph1*$^{-/-}$), < 0.0001 (5-HT, 3 dpi, *Tph1*$^{+/+}$ vs *Tph1*$^{-/-}$), 0.0232 (5-HT, 7 dpi, *Tph1*$^{+/+}$ vs *Tph1*$^{-/-}$). Source Data are provided as a Source Data file.

Lung - β diversity PCoA	Jaccard.PC1	*Tph1*$^{+/+}$ 0dpi *vs* *Tph1*$^{+/+}$ 3dpi	ns	ns
		Tph1$^{+/+}$ 0dpi *vs* *Tph1*$^{+/+}$ 7dpi	ns	ns
		Tph1$^{+/+}$ 3dpi *vs* *Tph1*$^{+/+}$ 7dpi	ns	ns
		Tph1$^{-/-}$ 0dpi *vs* *Tph1*$^{-/-}$ 3dpi	ns	ns
		Tph1$^{-/-}$ 0dpi *vs* *Tph1*$^{-/-}$ 7dpi	ns	ns
		Tph1$^{-/-}$ 3dpi *vs* *Tph1*$^{-/-}$ 7dpi	ns	ns
		Tph1$^{+/+}$ 0dpi *vs* *Tph1*$^{-/-}$ 0dpi	ns	ns
		Tph1$^{+/+}$ 3dpi *vs* *Tph1*$^{-/-}$ 3dpi	ns	ns
		Tph1$^{+/+}$ 7dpi *vs* *Tph1*$^{-/-}$ 7dpi	ns	ns
	Jaccard.PC2	*Tph1*$^{+/+}$ 0dpi *vs* *Tph1*$^{+/+}$ 3dpi	*	0.036
		Tph1$^{+/+}$ 0dpi *vs* *Tph1*$^{+/+}$ 7dpi	ns	ns
		Tph1$^{+/+}$ 3dpi *vs* *Tph1*$^{+/+}$ 7dpi	ns	ns
		Tph1$^{-/-}$ 0dpi *vs* *Tph1*$^{-/-}$ 3dpi	ns	ns
		Tph1$^{-/-}$ 0dpi *vs* *Tph1*$^{-/-}$ 7dpi	ns	ns
		Tph1$^{-/-}$ 3dpi *vs* *Tph1*$^{-/-}$ 7dpi	ns	ns
		Tph1$^{+/+}$ 0dpi *vs* *Tph1*$^{-/-}$ 0dpi	*	0.036
		Tph1$^{+/+}$ 3dpi *vs* *Tph1*$^{-/-}$ 3dpi	ns	ns
		Tph1$^{+/+}$ 7dpi *vs* *Tph1*$^{-/-}$ 7dpi	ns	ns

Lung- β diversity PCoA	Bray-Curtis.PC1	*Tph1*$^{+/+}$ 0dpi *vs* *Tph1*$^{+/+}$ 3dpi	ns
		Tph1$^{+/+}$ 0dpi *vs* *Tph1*$^{+/+}$ 7dpi	ns
		Tph1$^{+/+}$ 3dpi *vs* *Tph1*$^{+/+}$ 7dpi	ns
		Tph1$^{-/-}$ 0dpi *vs* *Tph1*$^{-/-}$ 3dpi	ns
		Tph1$^{-/-}$ 0dpi *vs* *Tph1*$^{-/-}$ 7dpi	ns
		Tph1$^{-/-}$ 3dpi *vs* *Tph1*$^{-/-}$ 7dpi	ns
		Tph1$^{+/+}$ 0dpi *vs* *Tph1*$^{-/-}$ 0dpi	ns
		Tph1$^{+/+}$ 3dpi *vs* *Tph1*$^{-/-}$ 3dpi	ns
		Tph1$^{+/+}$ 7dpi *vs* *Tph1*$^{-/-}$ 7dpi	ns
	Bray-Curtis.PC2	*Tph1*$^{+/+}$ 0dpi *vs* *Tph1*$^{+/+}$ 3dpi	ns
		Tph1$^{+/+}$ 0dpi *vs* *Tph1*$^{+/+}$ 7dpi	ns
		Tph1$^{+/+}$ 3dpi *vs* *Tph1*$^{+/+}$ 7dpi	ns
		Tph1$^{-/-}$ 0dpi *vs* *Tph1*$^{-/-}$ 3dpi	ns
		Tph1$^{-/-}$ 0dpi *vs* *Tph1*$^{-/-}$ 7dpi	ns
		Tph1$^{-/-}$ 3dpi *vs* *Tph1*$^{-/-}$ 7dpi	ns
		Tph1$^{+/+}$ 0dpi *vs* *Tph1*$^{-/-}$ 0dpi	ns
		Tph1$^{+/+}$ 3dpi *vs* *Tph1*$^{-/-}$ 3dpi	ns
		Tph1$^{+/+}$ 7dpi *vs* *Tph1*$^{-/-}$ 7dpi	ns

Feces - β diversity PCoA	Jaccard.PC1	*Tph1*$^{+/+}$ 0dpi *vs* *Tph1*$^{+/+}$ 3dpi	ns	ns
		Tph1$^{+/+}$ 0dpi *vs* *Tph1*$^{+/+}$ 7dpi	ns	ns
		Tph1$^{+/+}$ 3dpi *vs* *Tph1*$^{+/+}$ 7dpi	ns	ns
		Tph1$^{-/-}$ 0dpi *vs* *Tph1*$^{-/-}$ 3dpi	ns	ns
		Tph1$^{-/-}$ 0dpi *vs* *Tph1*$^{-/-}$ 7dpi	ns	ns
		Tph1$^{-/-}$ 3dpi *vs* *Tph1*$^{-/-}$ 7dpi	ns	ns
		Tph1$^{+/+}$ 0dpi *vs* *Tph1*$^{-/-}$ 0dpi	*	0.048
		Tph1$^{+/+}$ 3dpi *vs* *Tph1*$^{-/-}$ 3dpi	*	0.048
		Tph1$^{+/+}$ 7dpi *vs* *Tph1*$^{-/-}$ 7dpi	*	0.048
	Jaccard.PC2	*Tph1*$^{+/+}$ 0dpi *vs* *Tph1*$^{+/+}$ 3dpi	ns	ns
		Tph1$^{+/+}$ 0dpi *vs* *Tph1*$^{+/+}$ 7dpi	ns	ns
		Tph1$^{+/+}$ 3dpi *vs* *Tph1*$^{+/+}$ 7dpi	ns	ns
		Tph1$^{-/-}$ 0dpi *vs* *Tph1*$^{-/-}$ 3dpi	*	0.05
		Tph1$^{-/-}$ 0dpi *vs* *Tph1*$^{-/-}$ 7dpi	ns	ns
		Tph1$^{-/-}$ 3dpi *vs* *Tph1*$^{-/-}$ 7dpi	*	0.029
		Tph1$^{+/+}$ 0dpi *vs* *Tph1*$^{-/-}$ 0dpi	ns	ns
		Tph1$^{+/+}$ 3dpi *vs* *Tph1*$^{-/-}$ 3dpi	ns	ns
		Tph1$^{+/+}$ 7dpi *vs* *Tph1*$^{-/-}$ 7dpi	*	0.048

Feces - β diversity PCoA	Bray-Curtis.PC1	*Tph1*$^{+/+}$ 0dpi *vs* *Tph1*$^{+/+}$ 3dpi	0.029
		Tph1$^{+/+}$ 0dpi *vs* *Tph1*$^{+/+}$ 7dpi	0.029
		Tph1$^{+/+}$ 3dpi *vs* *Tph1*$^{+/+}$ 7dpi	ns
		Tph1$^{-/-}$ 0dpi *vs* *Tph1*$^{-/-}$ 3dpi	0.013
		Tph1$^{-/-}$ 0dpi *vs* *Tph1*$^{-/-}$ 7dpi	ns
		Tph1$^{-/-}$ 3dpi *vs* *Tph1*$^{-/-}$ 7dpi	ns
		Tph1$^{+/+}$ 0dpi *vs* *Tph1*$^{-/-}$ 0dpi	0.029
		Tph1$^{+/+}$ 3dpi *vs* *Tph1*$^{-/-}$ 3dpi	0.036
		Tph1$^{+/+}$ 7dpi *vs* *Tph1*$^{-/-}$ 7dpi	0.036
	Bray-Curtis.PC2	*Tph1*$^{+/+}$ 0dpi *vs* *Tph1*$^{+/+}$ 3dpi	0.048
		Tph1$^{+/+}$ 0dpi *vs* *Tph1*$^{+/+}$ 7dpi	0.048
		Tph1$^{+/+}$ 3dpi *vs* *Tph1*$^{+/+}$ 7dpi	0.029
		Tph1$^{-/-}$ 0dpi *vs* *Tph1*$^{-/-}$ 3dpi	0.025
		Tph1$^{-/-}$ 0dpi *vs* *Tph1*$^{-/-}$ 7dpi	ns
		Tph1$^{-/-}$ 3dpi *vs* *Tph1*$^{-/-}$ 7dpi	0.029
		Tph1$^{+/+}$ 0dpi *vs* *Tph1*$^{-/-}$ 0dpi	ns
		Tph1$^{+/+}$ 3dpi *vs* *Tph1*$^{-/-}$ 3dpi	0.029
		Tph1$^{+/+}$ 7dpi *vs* *Tph1*$^{-/-}$ 7dpi	ns

Table S3. Wilcoxon multiple-comparison (BH adjustment) tests on beta diversity PCoA components relative to Figure 5b and d. ns: p.adj >0.05; *:0.01<p.adj<=0.05. ns, not statistically significant.

Tryptophan biosynthesis	Anthranilate synthase	EC:4.1.3.27	Chorismate → Antranilate
	Phosphoribosylanthranilate isomerase	EC:5.3.1.24	Phosphoribosyl anthranilate ↔ Carboxyphenylamino-deoxy-ribulose-phosphate
	Indole-3-glycerol-phosphate synthase	EC:4.1.1.48	Carboxyphenylamino-deoxy-ribulose-phosphate ↔ Indole Glycerol phosphate
	Tryptophan synthase	EC:4.2.1.20	Indole Glycerol phosphate ↔ Trp
Indoles pathway	Tryptophanase	EC.4.1.99.1	L-tryptophan → Indole
	Monoamine oxidase	EC.1.4.3.4	Tryptamine → indol-3-acetaldehyde
	Amidase	EC.3.5.1.4	Indole-3-acetamide → Indole acetate
	Aldehyde dehydrogenase	EC.1.2.1.3	Indole-3-acetaldehyde → Indole acetate
Kynurenine pathway	Kynureninase	EC.3.7.1.3	N-formil-kynurinene → Formil anthranilate<br>L-Kynurenine → Anthranilate<br>Hydroxy-L-kynurenine → Hydroxyl-anatranilate
	Kynurenine monooxygenase	EC.1.14.13.9	L-Kynurenine → Hydroxy-L-kynurenine
	Hydroxy-anthranilate dioxygenase	EC.13.11.6	Hydroxyl-anatranilate → Amino carboxymuconate semialdehyde
	Anthranilate monooxygenase	EC.14.14.8	Anthranilate → Hydroxyl-anatranilate

Table S4. ***KEGG enzyme of tryptophan metabolism***

Aanat	forward AGGAGTCTCAGCTTCTCCTAGT<br>reverse CCTGTGTAGTGTCAGCGACT;
Ahr	forward TCCATCCTGGAAATTCGAACC<br>reverse TCTTCATCCGTCAGTGGTCTC
Asmt	forward TTCGACCTCTCGCCCTTCAG<br>reverse GAACACGGTGACCTCGCTG
Cyp1a1	forward ACAGTGATTGGCAGAGATCG<br>reverse GAAGGGGACGAAGGATGAAT
Cyp1b1	forward TTCTCCAGCTTTTTGCCTGT<br>reverse TAATGAAGCCGTCCTTGTCC
Ddc	forward GAGCTGGACAATCCCGACAA<br>reverse GATCAGGGGCCGAAGATAGC
Maoa	forward TTCAGCGTCTTCCAATGGGAGCT<br>reverse TGCTCCTCACACCAGTTCTTCTC
Foxp3	forward CCCAGGAAAGACAGCAACCTTTT<br>reverse TTCTCACAACCAGGCCACTTG
Gata3	forward TCTGGAGGAGGAACGCTAATG<br>reverse GGCTGGAGTGGCTGAAGG
Haao	forward TTGGGGACAGCTATGAGACC<br>reverse GCTCCCACACATACGAGGTT
Ido1	forward CCCACACTGAGCACGGACGG<br>reverse GCCCTTGTCGCAGTCCCCAC
Il13	forward AGCTGAGCAACATCACACAA<br>reverse GAATCCAGGGCTACACAGAA
Il17	forward GCTCCAGAAGGCCCTCAGA<br>reverse AGCTTTCCCTCCGCATTGA
Kc	forward CCGCTCGCTTCTCTGTGC<br>reverse CTCTGGATGTTCTTGAGGTGAATC
Kmo	forward GGTCGCCTTCACCAGAATAA<br>reverse CTATCAGGGACCCAAGGACA
Pu.1	forward GGTCCTAACCCCTCCACCTA<br>reverse TCTGGCTGGTGAAGTCCTCT
Rorc	forward ACAACAGCAGCAAGTGATGG<br>reverse CCTGGATTTATCCCTGCTGA
Sert	forward CGCAGTTCCCAGTACAAGC<br>reverse CGTGAAGGAGGAGATGAGGT
Tbet	forward GGACGATCATCTGGGTCACATTGT<br>reverse GCCAGGGAACCGCTTATATG
Tdo2	forward ATGAGTGGGTGCCCGTTTG<br>reverse GGCTCTGTTTACACCAGTTTGAG
Tph1	forward TCAGCCGAGAACAGTTGAATG<br>reverse GTCTTTGAAGCCAGGGTGGT
Tph2	forward CCTGAATCCGCCTGAGAG<br>reverse GGTGAGAGCATCTGTCTAACT
β-actin	forward AGCCATGTACGTAGCCATCC<br>reverse CTCTCAGCTGTGGTGGTGAA;

Table S5. ***List of primers***
