# Supplementary material for: Bridging of host-microbiota tryptophan partitioning by the serotonin pathway in fungal pneumonia
Source: Nat Commun. 2023 Sep 16;14:5753. doi: 10.1038/s41467-023-41536-8 (PMC10505232; doi:10.1038/s41467-023-41536-8)
Supplement: Supplementary file 3 — Description of Additional Supplementary Files [file 41467_2023_41536_MOESM3_ESM.pdf]

Description of Additional Supplementary Files

File Name: Supplementary Data 1
Description: Contribution of sequence variants to the indicated Enzyme Commission number (EC number) in the lung.

File Name: Supplementary Data 2
Description: Contribution of sequence variants to the indicated Enzyme Commission number (EC number) in the feces.
