# Supplementary material for: Bridging of host-microbiota tryptophan partitioning by the serotonin pathway in fungal pneumonia
Source: Nat Commun. 2023 Sep 16;14:5753. doi: 10.1038/s41467-023-41536-8 (PMC10505232; doi:10.1038/s41467-023-41536-8)
Supplement: Supplementary file 6 — Reporting Summary [file 41467_2023_41536_MOESM6_ESM.pdf]

nature portfolio

Corresponding author(s): Luigina Romani

Last updated by author(s): Aug 21, 2023

Reporting Summary

Nature Portfolio wishes to improve the reproducibility of the work that we publish. This form provides structure for consistency and transparency in reporting. For further information on Nature Portfolio policies, see our Editorial Policies and the Editorial Policy Checklist.

Statistics

For all statistical analyses, confirm that the following items are present in the figure legend, table legend, main text, or Methods section.

n/a	Confirmed	
☐	☒	The exact sample size (*n*) for each experimental group/condition, given as a discrete number and unit of measurement
☒	☐	A statement on whether measurements were taken from distinct samples or whether the same sample was measured repeatedly
☐	☒	The statistical test(s) used AND whether they are one- or two-sided<br>*Only common tests should be described solely by name; describe more complex techniques in the Methods section.*
☒	☐	A description of all covariates tested
☐	☒	A description of any assumptions or corrections, such as tests of normality and adjustment for multiple comparisons
☐	☒	A full description of the statistical parameters including central tendency (e.g. means) or other basic estimates (e.g. regression coefficient) AND variation (e.g. standard deviation) or associated estimates of uncertainty (e.g. confidence intervals)
☐	☒	For null hypothesis testing, the test statistic (e.g. F, t, r) with confidence intervals, effect sizes, degrees of freedom and P value noted<br>*Give P values as exact values whenever suitable.*
☒	☐	For Bayesian analysis, information on the choice of priors and Markov chain Monte Carlo settings
☒	☐	For hierarchical and complex designs, identification of the appropriate level for tests and full reporting of outcomes
☐	☒	Estimates of effect sizes (e.g. Cohen's d, Pearson's r), indicating how they were calculated

Our web collection on statistics for biologists contains articles on many of the points above.

Software and code

Policy information about availability of computer code

Data collection	Real-time RT-PCR was performed using the CFX96 Touch Real-Time PCR detection system (Bio-Rad) and analyzed with Bio-Rad CFX Manager.<br>All images were acquired using a BX51 fluorescence microscope (Olympus) using the analysis image-processing software (Olympus).<br>Blots were acquired using a ChemiDoc XRS+ Imaging system (Bio-Rad). Absorbance and fluorimetric signals were measured by a Tecan Infinite 200 reader and data collected with i-control 1.10 (for Infinite reader).
Data analysis	Image Lab 5.1 software (Bio-Rad)<br>GraphPad software V.6.01<br>FASTQC (version 0.11.9) software<br>Cutadapt (version 2.8)<br>Qiime2 platform (version 2022.2)<br>R version 4.2.1 (2022-06-23) in RStudio 2022.02.3+492 “Prairie Trillium” Release (1db809b8323ba0a87c148d16eb84efe39a8e7785, 2022-05-20) for Ubuntu Bionic Mozilla/5.0 (Windows NT 10.0; Win64; x64; rv:108.0) Gecko/20100101 Firefox/108.0<br>Cytoscape 3.9.1

For manuscripts utilizing custom algorithms or software that are central to the research but not yet described in published literature, software must be made available to editors and reviewers. We strongly encourage code deposition in a community repository (e.g. GitHub). See the Nature Portfolio guidelines for submitting code & software for further information.

Data

Policy information about availability of data

All manuscripts must include a data availability statement. This statement should provide the following information, where applicable:

- Accession codes, unique identifiers, or web links for publicly available datasets
- A description of any restrictions on data availability
- For clinical datasets or third party data, please ensure that the statement adheres to our policy

The datasets generated and/or analysed during the current study have been deposited in the Sequence Read Archive (SRA) (https://www.ncbi.nlm.nih.gov/sra) under BioProject ID number PRJNA980163 (https://www.ncbi.nlm.nih.gov/bioproject/PRJNA980163). The following databases were used: Silva138, KEGG, and MetaCyc. Source data are provided with this paper.

Research involving human participants, their data, or biological material

Policy information about studies with human participants or human data. See also policy information about sex, gender (identity/presentation), and sexual orientation and race, ethnicity and racism.

Reporting on sex and gender	Sex and gender were not considered in the study design. However, sex information along with other variables (age, FEV1, CFTR genotype) was collected at the time of informed consent. Sex and gender were not considered relevant to this study focused on the regulation of immune and microbial metabolic functions in respiratory pneumonia.
Reporting on race, ethnicity, or other socially relevant groupings	Caucasian origin
Population characteristics	Patients with a proven diagnosis of cystic fibrosis whose characteristics have been described in Moretti, S., Renga, G., Oikonomou, V. et al. A mast cell-ILC2-Th9 pathway promotes lung inflammation in cystic fibrosis. Nat Commun 8, 14017 (2017). https://doi.org/10.1038/ncomms14017.
Recruitment	Ten patients were selected for serotonin levels measurement based on comparable age (mean ± SD = 12.6 ± 1.9; range 10-16 years), a 1:1 male/female ratio, and all carrying the ΔF508 homozygous mutation.
Ethics oversight	Human studies approval was obtained from institutional review boards the Bambino Gesù Children's Hospital (Rome, Italy), Ospedale Maggiore Policlinico, University of Milan, (Milan, Italy), Innsbruck Medical University, (Innsbruck, Austria) and Servizio di Supporto Fibrosi Cistica, (Cerignola, Foggia, Italy).

Note that full information on the approval of the study protocol must also be provided in the manuscript.

Field-specific reporting

Please select the one below that is the best fit for your research. If you are not sure, read the appropriate sections before making your selection.

☒ Life sciences ☐ Behavioural & social sciences ☐ Ecological, evolutionary & environmental sciences

For a reference copy of the document with all sections, see nature.com/documents/nr-reporting-summary-flat.pdf

Life sciences study design

All studies must disclose on these points even when the disclosure is negative.

Sample size	Sample size was estimated based on our previous publications with similar experimental approaches (Moretti, S., Renga, G., Oikonomou, V. et al. A mast cell-ILC2-Th9 pathway promotes lung inflammation in cystic fibrosis. Nat Commun 8, 14017 (2017). https://doi.org/10.1038/ncomms14017).
Data exclusions	No data were excluded from the analysis
Replication	The data reported consisted of at least two independent experiments with similar results.
Randomization	Animals were randomly assigned to either the control or the experimental group. Littermates were used as controls, wherever possible. Appropriate controls are used in each experiment.
Blinding	The investigators were not blinded to allocation. However, experiments were performed without any prior biases, and conclusions made based on statistical significance of the data.

Reporting for specific materials, systems and methods

We require information from authors about some types of materials, experimental systems and methods used in many studies. Here, indicate whether each material, system or method listed is relevant to your study. If you are not sure if a list item applies to your research, read the appropriate section before selecting a response.

Materials & experimental systems

n/a	Involved in the study
☐	☒ Antibodies
☐	☒ Eukaryotic cell lines
☒	☐ Palaeontology and archaeology
☐	☒ Animals and other organisms
☒	☐ Clinical data
☒	☐ Dual use research of concern
☒	☐ Plants

Methods

n/a	Involved in the study
☒	☐ ChIP-seq
☒	☐ Flow cytometry
☒	☐ MRI-based neuroimaging

Antibodies

Antibodies used

Serotonin antibody (clone 5HT-H209, NOVUS Biologicals, NB120-16007V2, dil 1:50), Pgp9.5 antibody (clone EPR4118, Abcam, ab108986, dil 1:125), Chymase polyclonal antibody (Bioss, bs-2353R, dil 1:100), AhR antibody (clone RPT1, Invitrogen, AB_2273723, dil 1:100), AhR polyclonal antibody (Proteintech, 17840-1-AP, dil 1:1000), IDO1 polyclonal antibody (Millipore, AB9900, dil 1:500), CD41 polyclonal antibody (Emfret, M025-1, dil 1:200), alpha actin polyclonal antibody (Sigma-Aldrich, A2066, dil 1:200), beta tubulin antibody (clone D-10, Santa Cruz Biotechnology, sc-5274, dil 1:500), GAPDH antibody (clone 6C5, Santa-Cruz Biotechnology, sc-32233, dil 1:250).

Validation

Each antibody has been validated by the suppliers (see manufacturer's website). Additionally, most antibodies (anti-Chymase, -AhR, -IDO1, -alpha actin, -beta tubulin and -GAPDH) were used and validated in our previous publications (Moretti, S., Renga, G., Oikonomou, V. et al. A mast cell-ILC2-Th9 pathway promotes lung inflammation in cystic fibrosis. Nat Commun 8, 14017 (2017); Romani, L., Oikonomou, V., Moretti, S. et al. Thymosin α1 represents a potential potent single-molecule-based therapy for cystic fibrosis. Nat Med 23, (2017).

Eukaryotic cell lines

Policy information about cell lines and Sex and Gender in Research

Cell line source(s)

Mouse hepatoma H1L6.1c3 (containing pGudLuc6.1) cells.

Authentication

Authentication was performed in Van Langenhove K, Croes K, Denison MS, Elskens M, Baeyens W. The CALUX bio-assay: analytical comparison between mouse hepatoma cell lines with a low (H1L6.1c3) and high (H1L7.5c1) number of dioxin response elements. Talanta. 2011 Sep 30;85(4):2039-46. doi: 10.1016/j.talanta.2011.07.042.

Mycoplasma contamination

Cell lines tested negative for mycoplasma contamination.

Commonly misidentified lines
(See ICLAC register)

No commonly misidentified cell lines were used.

Animals and other research organisms

Policy information about studies involving animals; ARRIVE guidelines recommended for reporting animal research, and Sex and Gender in Research

Laboratory animals

6–to 8–wk–old of the following strains:
Tph1+/+ and Tph1—/— mice;
C57BL/6 mice;
C57BL/6-KitW/W-v;
B6.129-Ahrtm1Bra/J Ahr-deficient (Ahr—/—);
CF mice homozygous for the Phe508del-Cftr (Cftrtm1EUR, F508del) allele.
Mice were housed at a 12 light/12 dark cycle, with a temperature of 18-23°C and a 40-60% humidity.

Wild animals

The study did not involve wild animals

Reporting on sex

male and female

Field-collected samples

The study did not involve samples collected from the field

Ethics oversight

Mouse experiments were performed according to Italian Approved Animal Welfare Authorization 360/2015-PR and Legislative Decree 26/2014 regarding the animal license obtained by the Italian Ministry of Health lasting for 5 years (2015–2020) and the Welfare Authorizations 662/2020-PR, lasting for two years (2020–2022) and Legislative decree 26/2014 regarding the animal license, obtained by the Italian Ministry of Health.

Note that full information on the approval of the study protocol must also be provided in the manuscript.
